# The Effects of a Home-Based Combined Motor Control and Ergonomic Program on Functional Ability and Fear of Falling: A Randomized Controlled Trial

**DOI:** 10.7759/cureus.18330

**Published:** 2021-09-27

**Authors:** Sophia Stasi, Maria Tsekoura, John Gliatis, Vasiliki Sakellari

**Affiliations:** 1 Physiotherapy, Faculty of Health and Care Sciences, University of West Attica, Athens, GRC; 2 Physiotherapy, School of Health Rehabilitation Sciences, University of Patras, Patras, GRC; 3 Medicine, School of Health Sciences, University of Patras, Patras, GRC

**Keywords:** motor control exercise, physiotherapy interventions, fear of falling, falls, older people

## Abstract

Objectives

Physical exercise is a key intervention for improving functional ability and preventing falls in older people. However, the implemented interventions targeted balance, gait, and muscle strength, while little is known regarding motor control exercises in this population. Therefore, this study aimed to investigate the effects of a 12-week home-based motor control exercise program combined with an ergonomic home modification (the McHeELP program).

Patients and methods

Fifty-two older people (aged ≥65 years), who had experienced at least one fall incident in the past 12 months, were randomly assigned into two groups; the McHeELP group (McHeELP-G) (n=26) that received the McHeELP program and the control group (CG) (n=26). Physical performance measures (PPMs) and patient-reported outcomes (PROs) were used to evaluate participants. At baseline, 3rd month (post-intervention), and again at 6th month (follow up), balance control was assessed using the Tandem stance test (Tandem) and the Functional Reach Test (FRT). Functionality was assessed by the 4 meters walking test (4MWT), Timed Up and Go (TUG) test, 30 seconds-Sit to stand test and the Greek version of Lower Extremity Functional Scale (LEFS-Greek). The Greek version of the Falls Self-efficacy International scale (FES-I_GREEK) was used for the evaluation of "fear-of-falling" (FOF). The home falls and accidents screening tool (HOMEFAST) is used to identify home hazards. Two-way mixed ANOVA model, independent samples t-test, One-factor Repeated Measures ANOVA model and ANCOVA model were used for the statistical analysis of the data.

Results

Homogeneity was found between McHeELP-G and CG regarding the demographic and clinical characteristics, and no statistically significant difference was found at baseline measurements of PROs and PPMs, except HOMEFAST (p=0.031). Post-intervention (3rd month), the comparison of the absolute values between groups revealed that the McHeELP-G achieved statistically significant better balance control (longer Tandem stance test and higher values of FRT), better functionality [faster gait speed (4MWT), shorter TUG performance time, and a higher number of repetitions at 30 seconds-Sit to stand] (all p-values <0.05), while no difference was found for LEFS-Greek score (p=0.095), compared to CG. In addition, McHeELP-G reported lesser FOF than CG [lower FES-I_GREEK score (p=0.041)], and fewer home-hazards [lower HOMEFAST score (p=0.041)]. At follow up measurement (6th month), all PPMs scores of McHeELP-G, regarding balance control and functionality, were remained statistically significant (all p-values <0.005), and the FES-I_GREEK score (p=0.034), while no difference was found between groups for LEFS-Greek score (p=0.146) and HOMEFAST score (p=0.185). Sensitivity analysis (from baseline to 3rd and 6th month) revealed similar findings to the "comparison of the absolute values between groups" analysis. The within-group changes from baseline to 3rd month of McHeELP-G were statistically significant improved for all PPMs and PROs (all p-values <0.05), while in CG, statistical significant difference was found for TUG, FRT-right, and HOMEFAST (p<0.05). Those within-group changes were also preserved until 6th month.

Conclusions

The study's findings provide encouraging evidence that McHeELP program may increase functional ability and decrease FOF of older people. However, further research is required for a thorough understanding of the effect of McHeELP program.

## Introduction

Fall incidents are a serious health issue that can reduce older people's independence related to daily-life activities, and quality of life. At least 33% of all community-dwelling people aged over 65 years fall each year [1]. Fall may occur due to the age-related deterioration of balance, decreased lower limbs' muscular strength [2], or deficits in motor performance [3]. Older people's motor performance deficits include increased variability, difficulty in coordination, slowing of movement, as well as gait and balance difficulties [3]. It has been reported that motor performance deficits could be reduced through training specificity, which is the crucial element of motor learning [4]. However, little is known regarding implementing motor control exercises in this population, the fall prevention interventions targeting balance, gait, and muscle strength [1].

In addition, since most falls may occur at home, home-safety interventions also have a role in preventing these incidents [5]. These interventions include adaptations necessary for older people to make the living environment safe and accessible [5]. Environmental fall-related risk factors include poor lighting, slippery or uneven surfaces, lack of handrails, and poor footwear [6]. Reducing the home living environmental hazards and modifying potential risk factors minimized the incidence of home-related falls and their consequences [6]. However, more research is needed to explore the impact of home modifications on fall prevention.

Fear of falling (FOF) is a common fear amongst older people, and high levels of FOF can increase the risk of future falls [7]. Therefore, FOF is considered an important outcome measure for fall prevention and management [8]. In older adults, numerous exercise intervention studies (multifactorial, balance, muscle strength interventions) have reported their effect on fear of falling. However, information about the efficacy of motor control exercises on FOF is limited. Hence, in addition to improving balance and functionality, it is important to investigate whether motor control exercises can reduce FOF.

Up to our knowledge, research findings on fall prevention regarding motor control exercises are limited, while the proposed interventions included only a few motor-cognitive stepping exercises [9,10]. Additionally, information regarding home interventions is many and varied [5], suggesting a need to explore these delivery options' effectiveness further.

This study aimed to evaluate a home-based motor control exercise program combined with ergonomic suggestions for arrangements of the home environment (the McHeELP program). We hypothesized that the McHeELP program would improve functional ability and FOF and reduce potential fall-risk factors in older people's home environment.

## Materials and methods

Trial design

The present study was a parallel-group randomized controlled trial (Clinical Trial Identifier: ISRCTN15936467) conducted following the ethical principles stated in the Declaration of Helsinki and its later amendments [11]. Ethical approval was obtained from the University of Patras Ethics committee (University of Patras, Greece; Reference number: 9807/05/02/2020). The study conformed to the "Consolidated Standards of Reporting Trials" (CONSORT) 2010 Statement checklist of information to include when reporting a randomized trial [12].

Participants and procedures

Participants were recruited from two regions of Greece, namely Attica and Achaia. Before recruitment, each participant was screened for eligibility. Inclusion criteria for enrolment included age 65 years or more and at least one fall incident in the past 12 months. Exclusion criteria included: (i) cognitive impairment, (ii) lower-limb muscle weakness due to a central or peripheral neurological etiology, (iii) participants were told not to exercise by a physician, (iv) currently participating in an exercise program. Additional criteria for exclusion were, participants suffering from a vision or vestibular problem or had knee and ankle joint's restrictions that could affect the proper performance of either the exercises or the study's selected physical performance measures (PPMs) [13]. Participants gave their written informed consent upon acceptance, and their demographic and clinical characteristics were recorded in a face-to-face interview [14]. The participants were allocated into either the intervention group (McHeELP-G) or Control group (CG) and were blinded to group allocation. The 1:1 allocation ratio was used for randomization [15].

Outcome measures and procedures

Participants were assessed three times in the present study: baseline, the end of 3rd month (post-intervention), and the end of 6th month (follow up). PPMs and patient-reported outcomes (PROs) were used to evaluate participants. The balance control was assessed by the Tandem stance (heel-toe) test (Tandem) [16] and the Functional Reach Test (FRT) [17]. Functionality was assessed using the 4 meters walking test (4MWT) [18], the Timed Up and Go (TUG) test [19], the 30 seconds - Sit to stand test [20], and the Greek version of Lower Extremity Functional Scale (LEFS-Greek) [21]. The Greek version of the Falls Self-efficacy International Scale (FES-I_GREEK) [8] was used for the evaluation of "fear-of-falling" (FOF), defined as ongoing concern about falling that ultimately limits the performance of activities of daily living (ADL) [22]. The home falls and accidents screening tool (HOMEFAST) [23] was used to identify hazards in older people's home. The description and procedures of the used outcome measures are presented below:

Tandem Test

The test requires the participant to maintain balance while standing in a tandem heel-to-toe position. The participant places the foot immediately in front of the other foot (heel to toe), arms down by their side. The time that the participant can hold the tandem stance is recorded in seconds. The lower limit value for tandem stance test is 10 seconds; a maximum of 30 seconds distinguishes community-dwelling older people of higher ability [16].

Functional Reach Test (FRT)

The participant, barefoot and standing upright, is positioned with one side (e.g. right) of the body close to the wall. A yardstick is attached to a wall at about shoulder height. The instruction is to "reach forward along the yardstick as far as you can without taking a step" along the yardstick. The location of the 3rd metacarpal is recorded. In centimetre (cm), the researcher measures the distance the person can reach forward beyond arm length while standing in a fixed position. A distance of less than 15 cm in FRT has been associated with an increased risk for fall in elderly people [17]. In the present study, both FRT-right and FRT-left were separately recorded.

4 Meters Walking Test (4MWT)

Participants are informed to walk 4 m at their usual speed. Gait speed is assessed in seconds by a manual chronometer. Timing starts at the first foot movement and ends when a foot completely crosses the finish line-the performance time recorded in seconds. Gait speed was calculated as distance in meters divided by time in seconds. In older people, gait speeds that is lower than 0.7 m/sec, indicating a high risk of falls and other adverse events, while gait speed that is either equal or above 1.1m/sec indicated high functioning [18].

Timed Up and Go (TUG) Test

Participants are seated in a standard 45 cm height chair, with the back against the chair, both arms resting along their body, and both feet completely resting on the floor. The TUG assesses the number of seconds needed for an individual to stand up from a chair, walk 3 meters at their usual pace past a line on the floor, turn around, walk back to the chair, and sit down again with the back against the chair. The performance time is recorded in seconds. The risk of falls increases as the TUG performance time increases. Scores of less than 10 seconds are consistent with independence in activities of daily living (ADL), in the older people [19].

30 Seconds - Sit to Stand Test

It records the number of stands a person can complete in 30-seconds. The test is administered using a chair without arms, with a seat height of 43 cm (17 inches). The number of stands is recorded. Regarding older people, aged 75-79, like our sample, the criterion standard to maintain functional independence is 13-14 stands, which is considered a cut-off score for moderately active older people [20].

LEFS - Greek

The Lower Extremity Functional Scale is a 20-item functional status questionnaire that aims to assess functional status and investigate the degree of difficulty a patient experiences in performing everyday tasks. The minimum score of zero corresponds to disability/very low functionality, while the maximum score of 80 indicates very high functionality) [21].

FES-I_GREEK

The Falls Self-efficacy International Scale is a 16-item questionnaire has been widely used assessing FOF. The score ranges from 16 to 64 points. Higher values indicate more concern about falling and less fall-related self-efficacy [8].

HOMEFAST

The home falls and accidents screening tool is used to identify hazards in older people's homes. It includes 25 items focusing on seven main areas of potential hazards: floors, furniture, lighting, bathroom, storage, stairways/steps and mobility. The score ranges from 0 to 25 points. The maximum score of 25 corresponds to a higher risk of falling within the home environment [23].

When collecting data, the interview survey and PROs interspersed with the PPMs. This process reduced the risk of question-order bias and allowed sufficient resting time between the tests. The PROs were given out in random order, and PPMs were performed only once to avoid affecting the participant's performance and minimize habituation bias. The measurement data were collected at the participants' home due to the needed equipment for the performance of the objective PPMs was portable. The measurements were carried out by two examiners (senior physiotherapists); one for Attica and one for the Achaia groups, which were blinded concerning the group assignment, and they did not involve in any other part of the study. During the study period (6 months), cases of a new fall-incident were also recorded [13].

Interventions

Intervention Group/McHeELP-G

Participants in this group received a home-based motor control exercise program combined with an ergonomic home modification for 12 weeks. The McHeELP exercise program includes a domain of “warm-up” exercises and five domains of motor control exercises, namely: "Serial skills", "Cognitive skills", "Balance", "Sensory strategy", and "Dynamic control". The concept of McHeELP exercise program, the details of the implementation, sets of repetitions, sessions' frequency, and progression of exercises are descripted in the published methodology report [14]. At baseline session, the physiotherapist provided the participants with a health and wellness education session and their individualized McHeELP program, while also trained them on how to perform exercises safely and correctly. After that, the physiotherapist revised the individuated program at three more time points (at the end of the 1st, 4th and 8th week) to make progressive adjustments to the exercises.

Control group/CG

The participants of this group received no exercises. This group, as the McHeELP-G, received the same health and wellness education session at baseline. Participants were instructed not to participate in additional exercising for the same period (12 weeks).

Regarding the McHeELP-home modification part, a booklet was provided to the participants in both groups on the baseline session, which included essential tips and advice on modifying their home's exterior and interior. The adjustments were low-cost, such as moving furnishers to create proper pathways, removing worn-out carpets or loose/deep piles, replacing lamps with insufficient lighting, fixing slippery surfaces [14]. The critical difference between McHeELP-G and CG was that the participants of CG were merely advised on their baseline session. In contrast, during the three intermediate appointment sessions, the participants of McHeELP-G were reminded to materialize these modifications.

Statistical analysis

The sample size estimation, which was calculated in our pilot study [13], showed that a total of 50 patients (25 patients per group) was required in order to have a 90% probability of demonstrating a between treatment difference of >15% (McHeELP-G: 32%±16 versus CG: 17%±16) in % change from baseline to end of 3rd month (the end of intervention) of Tandem variable with a significance of <5% (two-tailed test).

Data were expressed as mean±SD or median (IQR), in case of violation of normality, for continuous variables and as frequencies (percentages) for categorical variables. The Kolmogorov-Smirnov test was utilized for normality analysis of the continuous variables.

Homogeneity between groups was performed using the independent samples t-test or Mann-Whitney test in case of violation of normality for continuous variables and Chi-square test or Fisher's exact for categorical variables.

We used the two-way mixed ANOVA model using as factors "the intervention" (between groups) and "time" (within-group) for the analysis of all parameters. Since there was a statistically significant interaction between these factors for almost all variables, we used univariate analysis e.g. the comparison between groups for each time point separately and the comparison of time points for each group separately, making the appropriate adjustment of the p-values based on Bonferroni correction. More specifically, one factor Repeated Measures ANOVA model was used to compare different time measurements of variables for each group, and the t-test for independent samples was used for the between-groups comparison, at each time point separately, making all adjustments of the p-values. Between-group differences at each time point were reported as mean differences together with their respective 95% CI.

Sensitivity analysis, concerning baseline-balance between the two groups, was performed using analysis of covariance model (ANCOVA) using the absolute change in the measures of interest (3rd and 6th month) as dependent variable, the group (McHeELP or Control) as factor and the baseline value of the measures as covariate.

All tests are two-sided; statistical significance was set at p < 0.05. All analyses were carried out using the statistical package SPSS version 21.00 (IBM Corporation, Somers, NY, USA).

## Results

**Figure 1:**
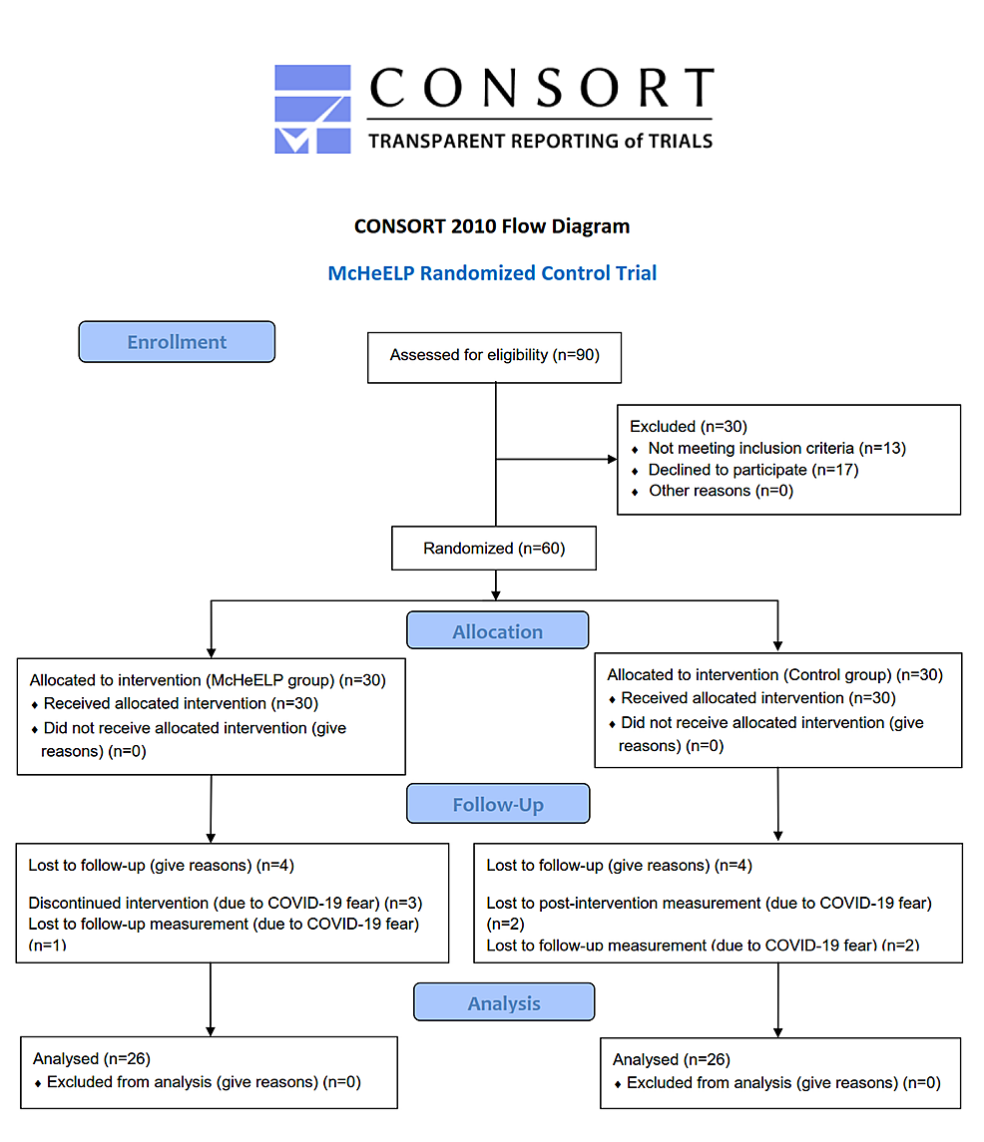
Flow diagram of the McHeELP randomized controlled trial. McHeELP: Motor Control Home Ergonomics Elderlies' Prevention of Falls; CONSORT: Consolidated Standards of Reporting Trials.

A total sample of 90 older people was assessed for eligibility, 13 were not meeting the inclusion criteria, and 17 people declined to participate. Finally, the data from 52 participants were analysed. The flow diagram in which the recruitment procedure is depicted is presented in Figure 1. Because of the low number of dropouts that were all unrelated to the intervention and the study's primary intention to evaluate the McHeELP program's efficacy, a per-protocol analysis was performed. 

The participants' demographic and clinical characteristics at baseline are shown in Table 1. The compared groups were homogeneous, since no statistically significant differences were found between groups. Similarly, no significant differences were found between the McHeELP-G and CG regarding the baseline measurements of the PROs and PPMs, except HOMEFAST (p=0.031) (Table 2). These findings proved the baseline-balance between groups.

**Table 1:** Demographic and clinical characteristics of the study’s sample (n=52). ^a^The values are presented as mean±standard deviation. ^b^The values are presented as median (interquartile range). McHeELP: Motor Control Home Ergonomics Elderlies' Prevention of Falls.

Characteristics	McHeELP group (n=26)	Control group (n=26)	p-value
Age (years)^a^	77.54±7.10	75.00±5.29	0.151
Gender [n(%)]			
Men	11(42.3)	9(34.6)	0.776
Women	15(57.7)	17(65.4)	
Height (meters)^a^	1.63±0.07	1.66±0.08	0.262
Weight (kilograms)^a^	76.31±11.35	77.08±9.33	0.792
BMI (kg/m^2^)^a^	29.27±3.65	27.64±3.94	0.228
Education [n(%)]			
Elementary	11(42.3)	10(38.5)	0.843
High school	10(38.5)	12(46,2)	
University	5(19.2)	4(15.4)	
Marital status [n(%)]			
Single/widowed	16(61.5)	13(50.0)	0.400
Married	10(38.4)	13(50.0)	
Number of children^b^	2(2.0)	1.0(2.0)	0.185
Living alone [n(%)]			
No	13(50.0)	14(53.8)	1.000
Yes	13(50.0)	12(46.2)	
Smoking [n(%)]			
No	24(92.3)	24(92.3)	1.000
Yes	2(7.7)	2(7.7)	
Alcohol [n(%)]			
No	17(65.4)	19(73.1)	0.764
Occasionally	9(34.6)	7(26.9)	
Sleeping hours [n(%)]			
7-8 hours	16(61,5)	17(65,4)	0.771
9-10 hours	10(38,5)	9(34,6)	
Number of daily meals^b^	3.5(1.0)	3.0(1.0)	0.107
Comorbidities^b^	4.0(1.3)	4.0(1.3)	0.342
Medications^b^	4.0(0.3)	4.0(2.0)	0.402
Surgery [n(%)]			
No	20(76.9)	19(73.1)	1.000
Yes	6(23.1)	7(26.9)	
Walking aid (stick) [n(%)]			
No	15(57.7)	19(73.1)	0.382
Yes	11(42.3)	7(26.9)	
Number of falls^b^	1.0(1.0)	1.0(1.0)	0.832
Falls injury [n(%)]			
No	22(84.6)	20(76.9)	0.726
Yes	4(15.4)	6(23.1)	
Concern about falling [n(%)]			
Somewhat	8(30.8)	8(30.8)	0.800
Fairly	9(34.6)	11(42.3)	
Very	9(34.6)	7(26.9)	
Ankle circumference^ a^	24.48±2.56	24.33±2.75	0.835

The Group x Time interaction test showed a significant interaction effect between "intervention" and "time" for all variables (all p values <0.05), except LEFS-Greek and HOMEFAST, which means that the difference between the interventions differs in each time point (Table 2).

**Table 2:** Comparison of clinical variables between groups at baseline and (Group x Time) interaction test. The values are presented as mean ±standard deviation. ^2^p-value based on F-Test for the Group x Time interaction from two-way mixed ANOVA model. LEFS-Greek: the Greek version of Lower Extremity Functional Scale [max =80 (very high functionality)]; FES–I­­­_GREEK: the Greek version of Falls Efficacy Scale –International [max=64 (severe fear of falling)]; HOMEFAST= the Greek version of Home Falls and Accidents Screening Tool [max=25 (higher risk of falling within home environment]; McHeELP: Motor Control Home Ergonomics Elderlies' Prevention of Falls.

Comparison of clinical variables between groups at baseline				(Group x Time) interaction test	
Clinical variables	McHeELP (n=26)	Control (n=26)	p-value	F values	p-value^2^
Tandem stance test (heel-toe)(seconds)	22.50±4.26	21.04±5.44	0.287	F(2.100)=10.17	p<0.005
4 meters walking test (meters/second)	1.0±0.21	0.97±0.18	0.475	F(2.100)=4.47	p=0.014
Timed Up and Go test(seconds)	12.10±1.59	12.62±1.44	0.218	F(2.100)=28.19	p<0.005
30 seconds – Sit-to-stand test (repetitions)	13.46±3.04	12.54±2.63	0.247	F(2.100)=4.61	p=0.012
Functional reach test – right (centimeters)	26.69±4.42	26.08±2.77	0.550	F(2.100)=14.73	p<0.005
LEFS–Greek	42.23±10.93	39.92±8.69	0.403	F(2.100)=1.10	p=0.335
FES–I_GREEK	41.65±10.17	39.77±9.94	0.502	F(2.100)=17.67	p<0.005
HOMEFAST	3.23±1.53	2.42±0.70	0.031	F(2.100)=0.35	p=0.706

Post-intervention, the comparison between groups of the absolute values of PPMs at 3rd month revealed that McHeELP-G achieved statistically significant longer Tandem test performance time (p<0.005), faster gait speed (4MWT) (p=0.005), shorter TUG test performance time (p<0.005), higher number of repetitions at 30 seconds-Sit to stand test (p=0.011), higher values of FRT-right (p<0.005), lower values of FES-I_GREEK score (p=0.041) and lower values of HOMEFAST score (p=0.041) compared to CG group. No difference was found between groups for LEFS-Greek score (p=0.095) (Table 3).

**Table 3:** Comparison of absolute values of clinical variables between groups during the observation period. The values are presented as mean±SD. ^1^All analyses based on Mixed ANOVA model, mean(95CI%). ^a^p<0.005 vs baseline, ^b^p<0.05 vs baseline, ^c^p<0.05 vs 3 months, ^d^p<0.005 vs 3 months (one-way repeated measures ANOVA). LEFS-Greek: the Greek version of Lower Extremity Functional Scale [max =80 (very high functionality)]; FES–I­­­_GREEK: the Greek version of Falls Efficacy Scale –International [max=64 (severe fear of falling)]; HOMEFAST= the Greek version of Home Falls and Accidents Screening Tool [max=25 (higher risk of falling within home environment]; McHeELP: Motor Control Home Ergonomics Elderlies' Prevention of Falls.

Clinical variables	Absolute values of 3^rd^ month						Absolute value of 6^th^ month			
	McHeELP (n=26)	Control (n=26)		Mean Difference^1^	p-value		McHeELP (n=26)	Control (n=26)	Mean difference^1^	p-value
Tandem stance test (heel-toe)(seconds)	28.19±4.13^a^		22.92±5.78	5.23(2.47/8.07)		<0.005	27.73±4.80^a^	22.46±5.88	5.27(2.28/8.26)	<0.005
4 meters walking test (meters/second)	1.25±0.22^ a^		0.95±0.20	0.3(0.93/0.14)		<0.005	1.18±0.22^a c^	1.0±0.22	0.18(0.62/0.26)	0.030
Timed Up and Go test (seconds)	8.48±1.78^ a^		11.77±2.20^b^	3.29(2.18/4.40)		<0.005	8.78±1.87^a^	11.56±2.41^b^	2.78(1.57/3.97)	<0.005
30 seconds – Sit-to-stand test (repetitions)	16.19±3.15^a^		13.69±3.67	2.50(0.59/4.41)		0.011	15.85±2.91^a^	13.54±3.47	2.30(0.53/4.10)	0.012
Functional reach test – right (cm)	35.27±3.76^a^		29.04±4.85^b^	6.23(3.81//8.65)		<0.005	34.38±3.97^a d^	28.31±4.93^b^	6.08(3.58/8.57)	<0.005
LEFS–Greek	45.19±9.93^b^		40.77±8.75	4.42(-0.79/9.64)		0.095	44.38±9.69	40.58±8.89	3.81(-1.37/8.98)	0.146
FES–I_GREEK	34.65±6.18^a^		39.15±9.00	4.50(0.20/8.80)		0.041	34.96±6.43^a^	39.62±8.76	4.65(0.37/ 8.93)	0.034
HOMEFAST	1.85±1.83^ a^		0.96±0.77^b^	0.89(-0.10/1.66)		0.042	1.77±1.82^a^	1.12±0.91^b^	0.65(-0.51/1.45)	0.185

The within-group changes from baseline to 3rd month of McHeELP-G were statistically significant improved for Tandem test, 4MWT, TUG test, 30 seconds-Sit to stand test, FRT-right, FES-I_GREEK score, HOMEFAST (p<0.005), and LEFS-Greek score (p<0.05), while in CG statistical significant difference was found for TUG, FRT-right and HOMEFAST (p<0.05) (Table 3).

In sensitivity analysis for baseline-balance, the comparison of absolute change from baseline to 3rd month of clinical variables between groups revealed similar findings compared to the absolute values between groups. Precisely, statistically significant difference in favour of McHeELP-G was found in Tandem test performance time (p<0.005), 4MWT (p=0.005), TUG test performance time (p<0.005), 30 seconds-Sit to stand test (p=0.013), FRT-right (p<0.005) and FES-I_GREEK score (p<0.005), while no statistically significant difference was found for the LEFS-Greek score, and the HOMEFAST (Table 4).

**Table 4:** Comparison of absolute change from baseline of clinical variables between groups during the observation period. The values are presented as mean (95%CI). All values are adjusted for baseline measurement estimated from ANCOVA model. LEFS-Greek: the Greek version of Lower Extremity Functional Scale [max=80 (very high functionality)]; FES–I­­­_GREEK: the Greek version of Falls Efficacy Scale –International [max=64 (severe fear of falling)]; HOMEFAST: the Greek version of Home Falls and Accidents Screening Tool [max=25 (higher risk of falling within home environment]; McHeELP: Motor Control Home Ergonomics Elderlies' Prevention of Falls.

Clinical variables	Absolute change from baseline to 3^rd^ month					Absolute change from baseline to 6^th^ month					
	McHeELP (n=26)	Control (n=26)	Mean difference	p-value	McHeELP (n=26)		Control (n=26)	Mean difference	p-value		
Tandem stance test (heel-toe)(seconds)	5.93(4.40/7.46)	1.64 (0.11/3.17)	4.29(2.12/6.46)	<0.005	5.43(3.82/7.03)		1.22 (-0.38/2.83)	4.20(1.90/6.49)		<0.005	
4 meters walking test (meters/second)	0.25(0.92/0.14)	0.02(0.52/0.05)	0.23(0.80/0.20)	<0.005	0.18(0.87/0.17)		0.03(0.48/0.03)	0.15(0.56/0.04)		0.031	
Timed Up and Go test (seconds)	-3.66(-4.26/-3.05)	-0.82(-1.43/-0.21)	2.84(1.97/3.71)	<0.005	-3.37(-4.07/-2.66)		-1.02(-1.73/-0.31)	2.35(1.33/3.35)		<0.005	
30 seconds – Sit-to-stand test (repetitions)	2.78(1.86/3.69)	1.11(0.20/2.02)	1.67(2.97/0.37)	0.013	2.49(1.55/3.43)		0.89(-0.46/1.83)	1.60(0.26/2.94)		0.020	
Functional reach test – right (centimeters)	8.81(7.11/10.51)	2.72(1.03/4.42)	6.10(3.69//8.49)	<0.005	7.95(6.18/ 9.72)		1.97(0.21/3.74)	5.98(3.47/8.48)		<0.005	
LEFS–Greek	3.20(1.17/5.22)	0.61(-1.41/2.64)	2.58(-0.29/5.45)	0.077	2.48(0.06/4.91)		0.32(-2.10/2.75)	2.16(-1.28/5.61)		0.233	
FES–I_GREEK	-6.68(-8.26/-5.10)	-0.94 (-2.53/0.65)	5.74(3.49/8.00)	<0.005	-6.36(-7.96/-4.78)		-0.48 (-2.08/1.12)	5.88(3.61/8.15)		<0.005	
HOMEFAST	-1.29(-1.72/-0.85)	-1.56(-2.0/-1.12)	0.27(-0.36/0.90)	0.404	-1.35(-1.82/-0.88)		-1.42(-1.89/-0.95)	0.07(-0.61/0.76)		0.834	

At follow up measurement, the comparison between groups of the absolute values of PPMs at 6th month revealed that McHeELP-G group in comparison to CG group had statistically significant longer Tandem test performance time (p<0.005), faster gait speed (4MWT) (p=0.030), shorter TUG test performance time (p<0.005), higher number of repetitions at 30 seconds-Sit to stand test (p=0.012), longer distance in FRT-right (p<0.005) and lower values of FES-I_GREEK score (p=0.034). No difference was found between groups for LEFS-Greek score (p=0.146) and HOMEFAST score (p=0.185) (Table 3).

The comparison between baseline and 6th-month measurements (within-group changes) showed, regarding McHeELP-G, a statistically significant difference improvement for all variables (p<0.005) while in CG statistical significant was found for TUG, FRT-right, and HOMEFAST (p<0.05). The 4 meters walking test and the FRT-right of McHeELP-G were also showed a significant difference between the 3rd and 6th month measurements (p<0.05 and p<0.005, respectively) (Table 3). 

In sensitivity analysis for baseline-balance, the comparison of absolute change from baseline to 6th month of clinical variables between groups revealed similar findings with the comparison of the absolute values between groups, as the 3rd month measurement. Statistically significant difference in favour of McHeELP-G was found in Tandem test performance time (p<0.005), 4MWT (p=0.031), TUG test performance time (p<0.005), 30 seconds-Sit to stand test (p=0.020), FRT-right (p<0.005) and FES-I_GREEK score (p<0.005), but no statistically significant difference was shown regarding the LEFS-Greek score, and the HOMEFAST score (Table 4).

Finally, no new incidents of falls were recorded during the study’s period (end of 6th month), in both groups.

## Discussion

In the present study, the implementation of the 12 weeks McHeELP program, which consisted of motor control exercises and ergonomic home modification significantly improved functionality and balance control, and lessened the FOF in older people who had a history of falls. The study's findings support our primary hypothesis. The post-intervention significant difference of the absolute values of the clinical variables of McHeELP-G was preserved until the follow-up (6th month). In addition, these results were also confirmed by sensitivity analysis.

The functional assessment results showed that post-intervention (3rd month), the McHeELP-G achieved statistically significant better balance control and functional performance than the CG, even though the baseline values of the PROs and PPMs of both groups were similar. Specifically, the Tandem test performance time of McHeELP-G reached close to the timeframe of 30 seconds that indicated high lateral postural stability that thought to be a sensitive key factor in preventing falls among older adults [24]. Compared to CG, the 4MWT value of McHeELP-G considered high, as it was above 1.1m/s. Gait speeds greater than 1.1m/s respond to outdoor activity independence, like crossing a pedestrian crossing with safety [24]. Regarding TUG performance time, McHeELP-G was under the cut-off score of 10 seconds, while CG was above. In this population, scores of less than 10 seconds are consistent with independence in activities of daily living (ADL) [19]. Finally, McHeELP-G accomplished more repetitions in the 30 seconds-Sit to stand than CG, a test that is one of the most biomechanically demanding functional tasks, also being essential for individual's independence [20]. It is worth noting that the changes in values of all PPMs and the statistical significance were maintained until the 6th month (follow up). On the other hand, although participants of McHeELP-C had a better LEFS-Greek score (approximately 4.5 points) compared to CG, a statistically significant difference did not accomplish. This could be explained because the minimum detectable change (MDC) for the LEFS is 9 points [25]. Nevertheless, our findings provide encouraging evidence for the effects of McHeELP exercise program on the overall functional ability, and they are in line with systematic reviews that it was reported that exercise training improved functional ability in older people [1,6,7]. The motor control exercises of McHeELP program might be a novel approach to fall prevention in older people.

Interestingly, the within-group comparison showed that participants of CG had improvement in TUG (-1 second) and FRT (3 centimetres) tests, even though they were not involved in any exercise program. We cannot explain these particular findings; however, the CG did not significantly improve the overall balance control and functionality during the study. The participants of McHeELP-G had a specific benefit from the program, not only in terms of TUG and FRT test results improvement, but also in Tandem stance test, 4MWT, and 30 seconds-Sit to stand, that is, in the overall functional ability. These findings further emphasize the effectiveness of McHeELP exercise program due to all the above PPMs reproduce movements and specific tasks related to ADLs, and as a whole reflect the level of the older people's functional independence [26]. 

In respect to FOF’s results, a statistically significant difference was observed post-intervention in the FES-I_GREEK between McHeELP-G and CG; this significant difference was maintained at follow-up (6th month). Since the study's participants were asked to assess their potential "fear-of-falling" using the FES-I_GREEK, our findings suggested that McHeELP program may be adequate to reduce the FOF of older people. In a recent systematic review, it was concluded that exercise interventions probably reduce FOF immediate post-intervention, but there was a not statistically significant effect in the longer-term period [<6 months (SMD 0.17, 95% CI −0.05, 0.38 (four studies) and ≥6 months post-intervention period (SMD 0.20, 95% CI −0.01, 0.41) (three studies)] [7]. In our study, the significant difference was maintained until the 6th month, which may be because McHeELP program incorporates both motor control exercises and home environment modifications. However, further research with a longer follow-up period is required for a thorough understanding of the effect of this particular intervention on the FOF.

In both groups, the McHeELP- home modification part reduced the potential fall-risk factors in the home's environment. Post-intervention (3rd month) a statistically significant difference between groups was found regarding HOMEFAST score, while at follow-up this difference was eliminated. In addition, the within-groups changes of both groups, at 3rd month, showed a statistically significant difference that was also preserved at the follow-up measurement. These findings were explained by the fact that participants of both groups made most of the McHeELP booklet's proposed home environment modifications, although CG was only advised on their baseline session to materialize them. A possible explanation might be that all our participants were fallers. Their fall-experience made them realize the potential risk and proceed to low-cost changes making their home environment less hazardous. Τhese home environment modifications could be the reason that no patient of both groups experienced a new fall-incident before the date of the follow-up measurement (end of 6th month). Education on home environment modifications is essential to maintain daily residential safety and could be preventing falls [27].

Most studies focus on centre-based exercise interventions, which may present accessibility challenges for older people, who may be unwilling or unable to travel and follow an outdoor program. Therefore, home-based exercise interventions may be a feasible alternative for this population by eliminating the challenge to the travel requirement [28]. In the prescription of home-based program, an initial challenge is to adapt the level of difficulty of the proposed exercises to each individual's capacity. Individualized exercise programs that are implemented at home may optimize the training's effectiveness, improve functionality and help lower the risk of falls for older people [28]. The McHeELP is a personalized exercise program that can be implemented at home. The exercises are chosen according to the personal's level of functional ability, musculoskeletal limitations and exercise responses. These criteria are considered the most essential exercise prescription for the elderly [29]. One more possible advantage of the McHeELP program is the ability to provide a well-structured comprehensive program without added special and expensive equipment and/or technology expenditures [14].

This study provides evidence that older people with a history of falls can safely and effectively participate in an individualized home-based program. The excellent feasibility and acceptability of McHeELP were confirmed in our pilot study [13]. This program may significantly contribute to physiotherapists and health practitioners by developing a novel evidence-based approach to fall prevention in older people.

Strengths and limitations

This study was a parallel-group randomized controlled trial (Clinical Trial Identifier: ISRCTN15936467). The participant's adherence was ensured by the supervision and guidance from the physiotherapist during the regular home visits and the telephone counselling between visits. Furthermore, all measurements were made by examiners, who were not involved in any other part of the study, and were blinded concerning the group assignment. These factors added strength and statistical power to the results of the present study.

On the other hand, there are significant limitations that must be mentioned. First, a limitation of this study is that our sample did not include a relatively diverse sample. Therefore, it must be underlined that our findings cannot be generalized to all fallers since older people with independent risk factors for falls such as visual impairment, dizziness or cognitive impairment were not included in our study's sample. Another limitation is that participants were followed up until the end of 6th month; therefore, it is unclear whether the observed post-intervention differences between McHeELP-G and CG will be maintained over time. Finally, regarding the participants' acceptability, a possible risk of bias may be that highly experienced physiotherapists in the geriatric population selected the appropriate exercises for the individualized home-based program, which may also influence the participants' compliance. Thus, the McHeELP program possibly cannot be implemented for every community-dwelling older adult, at least not without previews training of the trainers.

## Conclusions

The results shown here indicate that implementing a novel home-based and low-cost intervention may enhance functionality, improve balance and reduce FOF in community-dwelling older people. However, our findings should be interpreted with caution and more trials with longer follow up period are needed to strengthen the clinical evidence for McHeELP program. Exercise programs, easy-to-administer, need to be developed and implemented to reduce the incidents of falls.
